# Unsupervised feature extraction of anterior chamber OCT images for ordering and classification

**DOI:** 10.1038/s41598-018-38136-8

**Published:** 2019-02-04

**Authors:** Pablo Amil, Laura González, Elena Arrondo, Cecilia Salinas, J. L. Guell, Cristina Masoller, Ulrich Parlitz

**Affiliations:** 1 Universitat Politècnica de Catalunya, Rambla Sant Nebridi 22, 08222 Terrassa, Spain; 2 Instituto de Microcirugía Ocular, Josep Mar´ıa Lladó 3, 08035 Barcelona, Spain; 3 Max Planck Institute for Dynamics and Self-Organization, Am Faßberg 17, 37077 Göttingen, Germany

## Abstract

We propose an image processing method for ordering anterior chamber optical coherence tomography (OCT) images in a fully unsupervised manner. The method consists of three steps: Firstly we preprocess the images (filtering the noise, aligning and normalizing the resolution); secondly, a distance measure between images is computed for every pair of images; thirdly we apply a machine learning algorithm that exploits the distance measure to order the images in a two-dimensional plane. The method is applied to a large (~1000) database of anterior chamber OCT images of healthy subjects and patients with angle-closure and the resulting unsupervised ordering and classification is validated by two ophthalmologists.

## Introduction

Machine learning methods are extremely useful in biomedicine^1,2^ and in particular for glaucoma detection^3–6^. The use of such methods can help to optimize the available human resources, to increase accuracy in diagnosis, and to make treatment decisions faster.

Glaucoma is the leading cause of global irreversible blindness^7^. Early diagnosis and treatment is a challenge given that glaucoma presents no symptoms in its early stages^8^. Diagnostic of angle-closure is based on the clinical observation of the angle at the slit- lamp requiring a goniolens that is placed on the patient’s cornea. Anterior Segment Optical Coherence Tomography (AS-OCT) is a fast, useful and contact-less tool that allows visualization and measurement of the anterior chamber angle^9–11^. Various techniques exist and are being developed to improve image quality^12–15^, and work is also focused on the development of advanced tools to analyze such images^16–18^. However the quality of the images is not always enough for an accurate diagnosis and for example^19^, two glaucoma experts working together were unable to locate the scleral spur (see Fig. 8) in 28% of the images. Such landmark is of utmost importance to determine most of the relevant features that are used in angle-closure diagnosis.

Previous approaches for processing anterior chamber OCT images used segmentation algorithms and further analysis, while others used manual landmark determination. In works by Tian *et al*.^16^ and Wu *et al*.^20^ segmentation algorithms for OCT anterior chamber images were proposed, both discuss the difficulties of such task due to the noise in the image and to other artifacts. In^21^ the authors analyzed data from manual landmark determination to classify images into five subcategories of angle-closure glaucoma. They used both supervised and unsupervised feature selection and AdaBoost classifiers (supervised learning) to achieve accuracies in the range of 84~87%. In^22^ a method was proposed to extract almost 3000 features from the raw images, then, the most relevant features for classification were supervisedly selected, and machine learning was applied to the selected features. However, the fact that only 74 images were analyzed while 15 features were used, compromised the statistical significance of the study. In^23^ Histogram of Oriented Gradients (HOG) features of anterior chamber OCT images and Support Vector Machine (SVM, supervised learning) were used to classify different glaucoma subtypes, achieving accuracies in the order of 80%.

In this paper we propose an image processing method that *unsupervisedly* orders OCT anterior chamber images according to what we demonstrate to be relevant extracted features. The reliability of the algorithm is tested with a large number of images (~1000). Importantly, our method is fully autonomous and can be used to analyze images with a wide spectrum of quality, even those with high levels of noise and artifacts.

## Results

**Figure 1:**
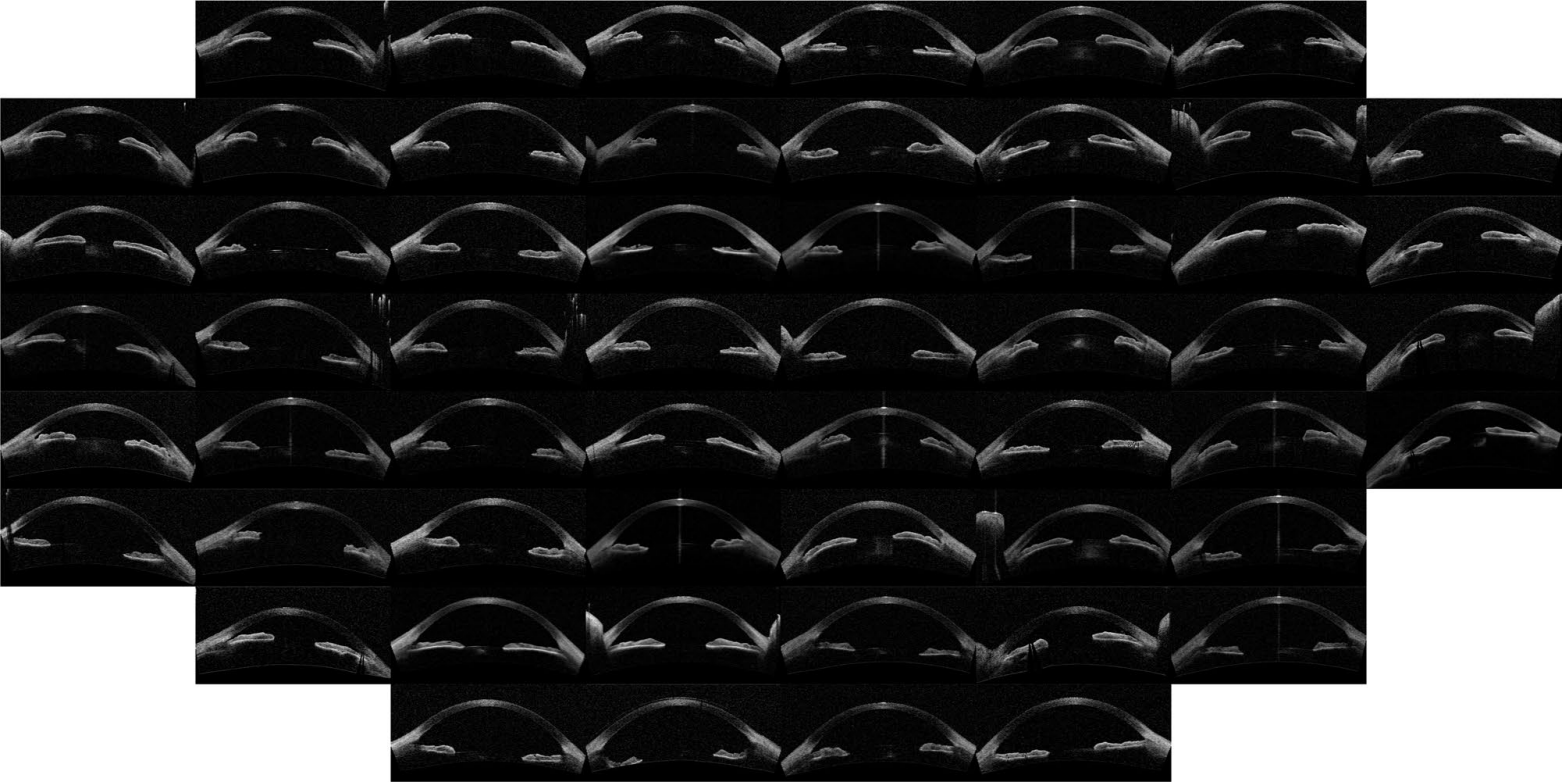
Image map obtained when using *IsoMap* and the Euclidean distance, without performing the alignment step in the pre-processing of the images.

**Figure 2:**
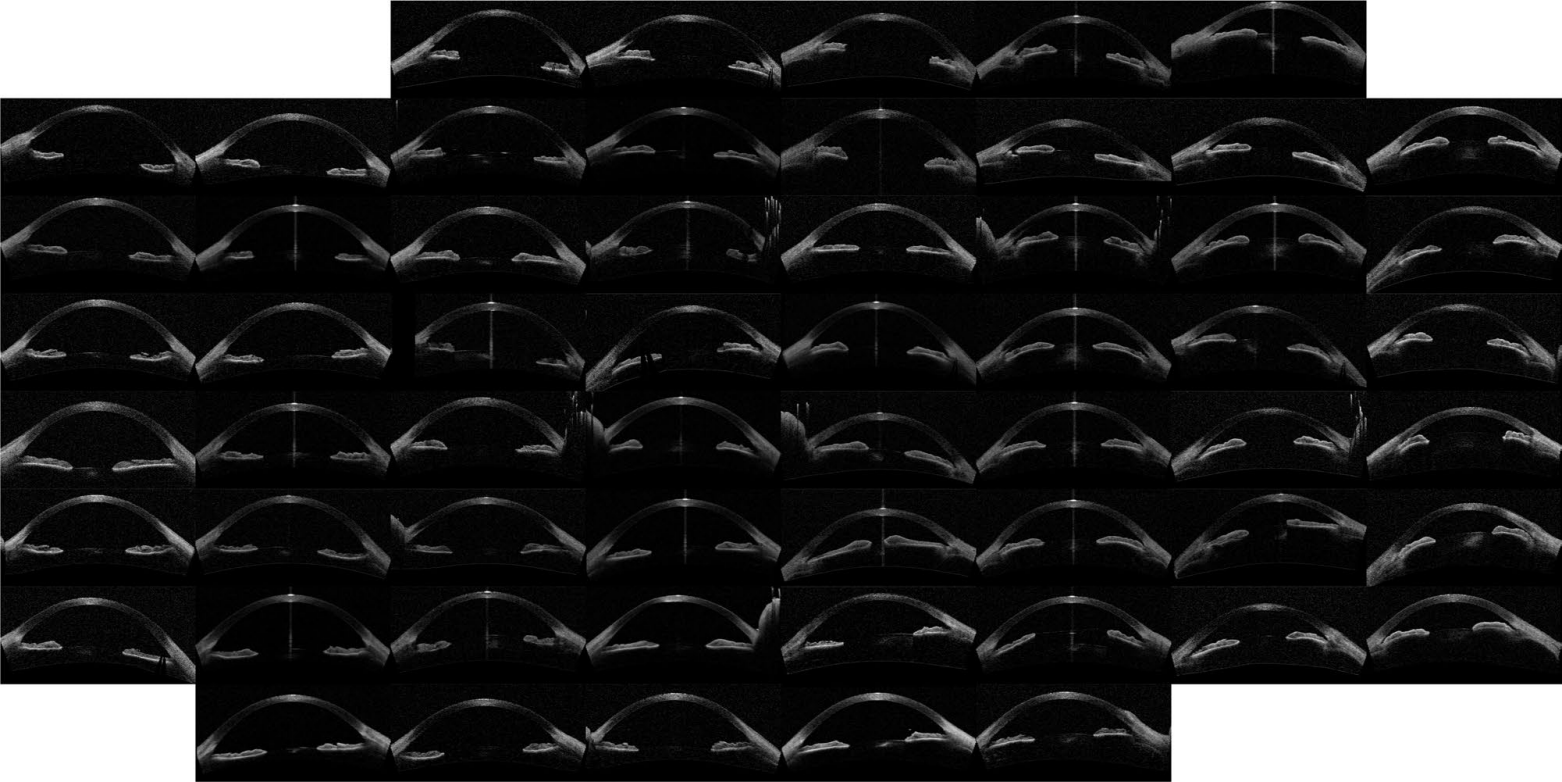
Image map with results using *IsoMap* and the Euclidean distance, including the alignment step in the pre-processing of the images.

**Figure 3:**
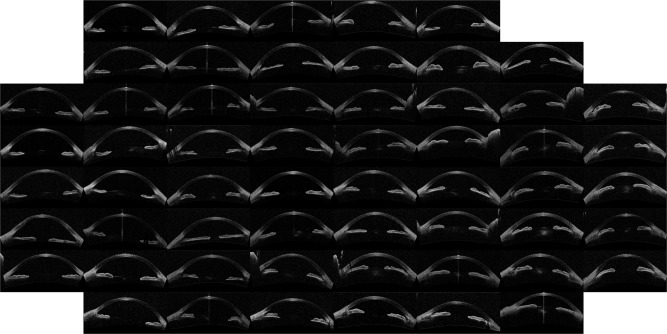
Image map with results using *IsoMap* and the Hellinger distance, including the alignment step in the pre-processing of the images.

**Figure 4:**
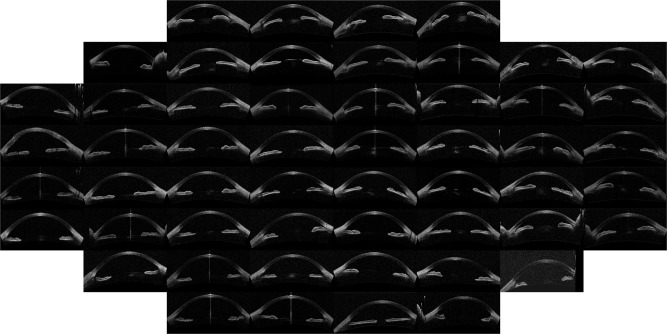
Image map with results using *t-SNE* and the Hellinger distance, including the alignment step in the pre-processing of the images.

The outcome of the algorithm applied to the image database (both described in Sec. *Methods*) is presented in the way of an “Image Map”. A regular grid in the coordinates space (*w*, *v*) is defined and one image per grid point is displayed. The results are presented in Figs 1–4 that show the Image Map obtained after applying *IsoMap* to the Euclidean distance (Fig. 1), to the aligned Euclidean distance (Eq. 4, Fig. 2), to the aligned Hellinger distance (Eq. 3, Fig. 3) and after applying *t-SNE* to the Hellinger distance (Fig. 4). These figures are ordered with increasing complexity and performance of the corresponding algorithm.

In Fig. 1 (*IsoMap* with Euclidean distance) it is apparent that the algorithm ordered the images according to the orientation (horizontal axis) and position (vertical axis) of each eye inside the OCT image, which are irrelevant features, this simple algorithm is, then, not capable of extracting useful features. However, when including the alignment step in the pre-processing of the images which removed the variability detected by the first algorithm, meaningful features were extracted, as it can be seen in the Image Map in Fig. 2 (these features correlate with the features derived manually, as shown in Table 1). A similar map was obtained with the Hellinger distance, shown in Fig. 3 which marginally improves the performance with the Euclidean distance. To test the robustness of the image ordering, the *t-SNE* algorithm was applied (instead of *IsoMap*). The map obtained is shown in Fig. 4, which turned out to perform slightly better than *IsoMap* according to the correlations shown in Table 1.

**Table 1:** Correlation coefficient between the automatic feature extracted (selecting the direction in the mapped space that gives the larger correlation) and the manual annotation features (mean angle and depth).

Method	Mean angle	Chamber Depth	Manual classification
IsoMap, no alignment	0.05	0.07	0.18
IsoMap, aligned euclidean	0.79	0.88	0.77
IsoMap, Hellinger	0.80	0.89	0.79
t-SNE, Hellinger	0.80	0.90	0.81

We also present the correlation with manual labels (0 corresponds to wide open, 1 to open, 2 to narrow, and 3 to closed). For comparison the correlation coefficients of the manual labels and anterior chamber depth is 0.8 while with ARA_500 (see^21^) is 0.75.

**Figure 5:**
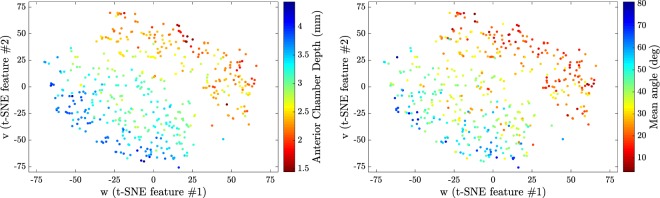
Left: Comparison between t-SNE results and chamber depth from manually annotated images. Right: Comparison between t-SNE results and mean angle from manually annotated images.

In Fig. 5 the features returned by the unsupervised algorithm, using *t-SNE*, are compared with the features obtained from the manual annotation of the images: in the left panel the color code indicates the chamber depth, and in the right panel, it indicates the mean angle (average of α and *β*) of each annotated image. Clearly, the features obtained from the manual annotation correlate very well with the features returned by the unsupervised algorithm. However, one should notice that the annotated features are not independent, but strongly correlated with each other. A summary of the correlation between the ordering (Mappings) and different features is shown in Table 1.

**Figure 6:**
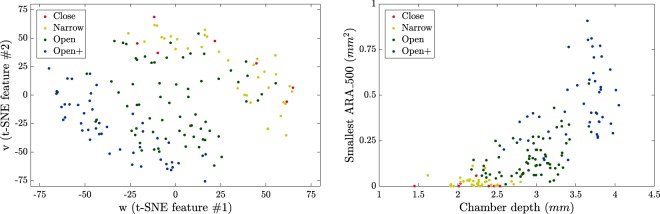
Left: Comparison between t-SNE results and manual classification. Right: Comparison between annotated results (angle and smallest ARA_500, Angle Recess Area see^21^) and manual classification.

Finally, the manual classification done by two expert ophthalmologists is included in the *t-SNE* (*w*, *v*) map. In Fig. 6, left panel, the color code indicates the different classes, and it can be noticed that the data points that represent images in the “wide-open” category are scattered in the left-down corner of the map, while the data points that represent images in the “closed” category are scattered in the right-top corner of the map.

In order to demonstrate that the separation between different classes obtained from features retrieved from the manual annotation of the images is comparable to the one obtained from the *t-SNE* features, the manual annotation features (mean angle and smallest ARA_500) are plotted in Fig. 6, right panel. A comparison with the left panel reveals that the manual annotation features also do not allow for a clear-cut separation of the different classes, and therefore, for classification purposes, the features obtained with the *t-SNE* map are as good as the manual annotation features.

## Discussion

We have proposed a new algorithm for ordering anterior chamber OCT images in such a way that it is possible to classify them, in a fully unsupervised manner, in meaningful groups according to relevant features. We have tested the algorithm with a large set of images classified by two expert ophthalmologists, and with a larger set of annotated images. We have verified that the separation in the different classes defined by the ophthalmologists (closed, narrow, open, and wide open) is similar when using the manually extracted features, or when using the features that are returned by the unsupervised algorithm (Fig. 6).

Therefore, the abstract features generated by the algorithm provide novel tools for assessing OCT images of the anterior chamber. They can be used for direct classification of the images and, furthermore, they can be linked to established quantities used for characterizing diseased eyes (like chamber depth, iris-corneal angle) resulting in an automatic detection system. As the algorithm is fully unsupervised, it can be easily automated and set up in OCT imaging systems to aid technicians and doctors in an early diagnosis.

The two main advantages of the algorithm demonstrated here over previous works are that it doesn’t need any ground truth or gold standard for training, and it does not rely on specific landmarks; thus, it can analyze images in which relevant landmarks are not visible or not easy to locate.

## Methods

### Data

The data consists of 1213 OCT images taken from consenting patients at IMO (Ocular Microsurgery Institute, Barcelona)^24^. The images were acquired using a Visante OCT instrument (Carl Zeiss Meditec) using the “Anterior segment” scan and the “Enhanced anterior segment” scan. The original resolution of all the images is 256-by-1024 pixels corresponding to an area of 16 mm-by-8 mm. All the patients included in the study consented that their data and images could be used for research and teaching purposes and the study has been approved by the ethical committee for clinic research in IMO. The patients were selected based on the already available data at IMO (retrospective study). We selected 247 patients at IMO database, 81 of which were glaucoma patents and the rest was a mixed group of healthy patients and patients with other diseases different from glaucoma and with normal intraocular pressure, 104 of those were cataract patients, from which we used images from before and after intraocular lens implant (images with intraocular lens implants were processed in the same way as the rest of the images). The average age of the subjects (at the time of the procedure) is 42 years and 57% of such subjects are female. We retrieved all the available images of the selected patients that corresponded to the mentioned scans. We had to discard around 1400 images because they didn’t depict the whole anterior segment (they were deliberately zoomed in) and around 50 due to very poor quality.

#### Manual annotation and classification of images

**Figure 7:**
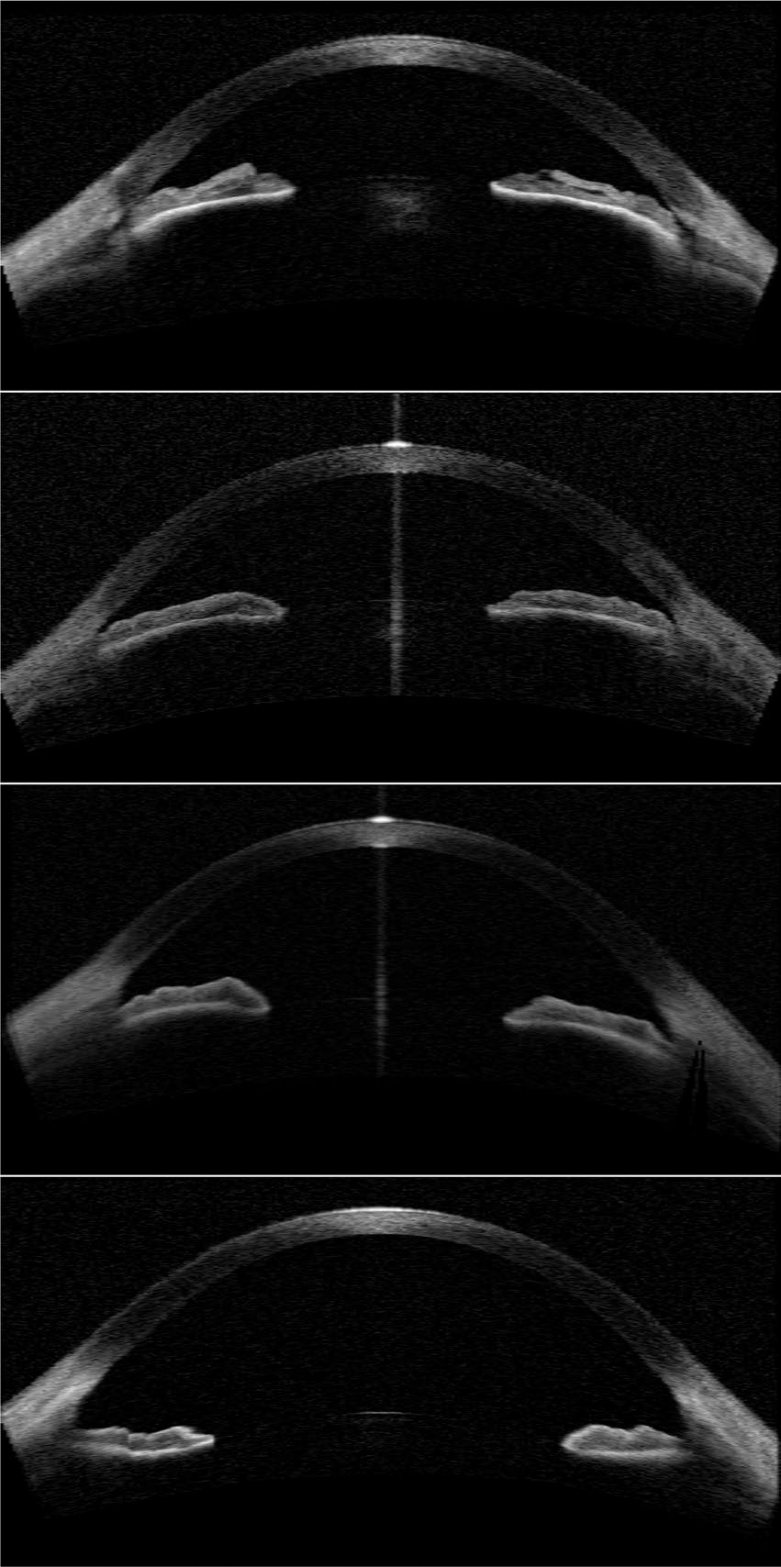
Example images of the categories classified by glaucoma experts. Closed (top), narrow (middle-top), open (middle-bottom), and wide open (bottom).

Two glaucoma experts (Elena Arrondo, MD and Cecilia Salinas, MD) evaluated a subset of 160 images and classified them into four categories: closed, narrow, open, and wide open. An image example in each category is displayed in Fig. 7. It is important to remark that this manual classification was not used by the algorithm (as it does not “learn” and thus, it does not require any training set); the manual classification was only used to test the relevance of the features returned by the nonlinear dimensionality reduction algorithms.

**Figure 8:**
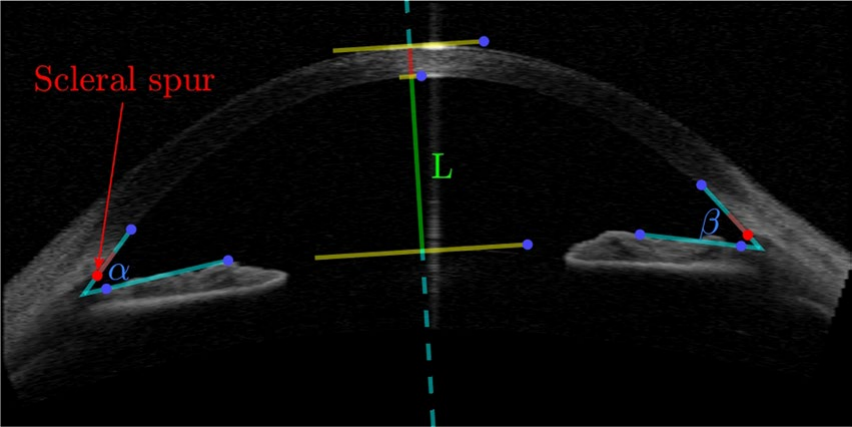
Example of an annotated image with landmarks and guidelines.

To test the relevance of the features extracted by the algorithm, several relevant landmarks were manually annotated in a subset (~400) of the images. The landmarks used are (see Fig. 8):

- Scleral spur.
- A second point near the scleral spur in the inside edge of the cornea (to set a line approximating the inside edge of the cornea).
- Two points on the top edge of the iris.
- Points in the inside and outside edge of the cornea in the center
- A point in the top edge of the lens in the center.
  From those landmarks the following features were calculated:
- Anterior chamber depth (L).
- Iris-corneal angles (*α* and *β*).
- Angle recess area (ARA_500, see^21^)

It has to be noted that some landmarks were not always clearly visible, in such cases the human expert guessed its position based on nearby features. The most common landmark that had to be guessed was the point in the top edge of the lens, which can be guessed based on the position of the iris. The sclerar spur is also frequently difficult to find but usually the clues were enough to guess its position by the expert. Ultimately, if the expert thought that it wasn’t possible to make a good guess, the image was simply skipped and omitted from the subset of manually annotated images. As with the classification, these landmarks were not used by the algorithm, rather they were used afterwards to evaluate its results.

### Unsupervised ordering and classification algorithm

In this section we present the algorithm for the unsupervised ordering and classification of OCT images. The input to the algorithm is a database of anterior chamber OCT images and the output is a map in a two dimensional plane. The algorithm performs three main steps: pre-processes the images, calculates a pair-wise distance measure between images and applies nonlinear dimensionality reduction.

#### Image pre-processing

The pre-processing of the images consists of the following substeps: homogenization, filtering, centering and aligning.

Homogenization: in each image, the intensity of each pixel was converted to double precision and normalized to be in between 0 and 1 (by linearly rescaling). Then, the horizontal and vertical spatial resolutions were adjusted to be the same (note that the original spacial resolution is anisotropic).

**Figure 9:**
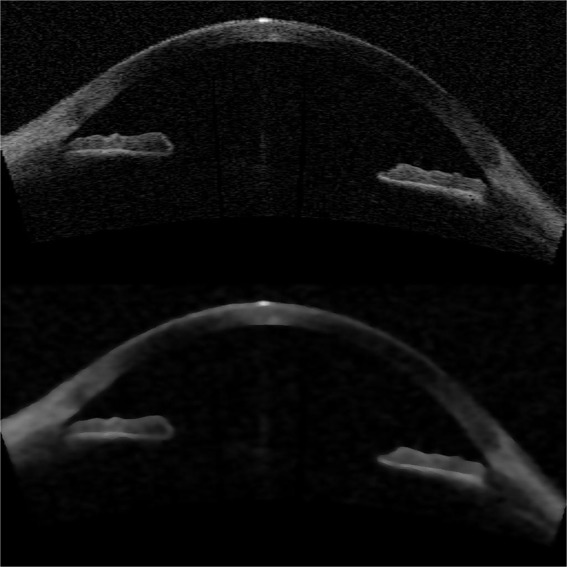
Example raw OCT image (top), and the same image after the filtering process (bottom).

Filtering: First, a two dimensional rectangular median filter was applied to the image (with a 0.055 mm-by-0.117 mm rectangle). This filter was needed because of the process of adjusting the spatial resolution, which resulted in the noise being spread more in one direction than in the other. Then, an anisotropic diffusion^25,26^ filter was applied to smooth the image, removing the spatial high-frequencies while preserving relevant edges. An example of such filtering is shown in Fig. 9.

For centering and aligning a set of statistical properties of the images were calculated, namely:

1 $$\begin{array}{llllll}S & = & \sum _{i,j}\,M(i,\,j), & X & = & \sum _{i,j}\,jM(i,\,j)\,,\\ Y & = & \sum _{i,j}\,iM(i,\,j), & XX & = & \sum _{i,j}\,{j}^{2}M(i,\,j)\,,\\ YY & = & \sum _{i,j}\,{i}^{2}M(i,\,j), & XY & = & \sum _{i,j}\,ijM(i,\,j)\,,\end{array}$$

where *M*(*i*, *j*) is the (gray) value of the image on the pixel that is *i* pixels down from the top edge and *j* pixels right from the left edge. With these quantities the centroid of the image (whose coordinates are $(i,\,j)=(\frac{Y}{S},\,\frac{X}{S})$) and the covariance matrix (COV) were calculated:

2 $$COV=(\begin{array}{cc}\frac{YY}{S}-\frac{{Y}^{2}}{{S}^{2}} & \frac{XY}{S}-(\frac{X}{S})(\frac{Y}{S})\\ \frac{XY}{S}-(\frac{X}{S})(\frac{Y}{S}) & \frac{XX}{S}-\frac{{X}^{2}}{{S}^{2}}\end{array})\,,$$

From the covariance matrix the eigenvector *v*_1_, corresponding to the largest eigenvalue, was calculated and used to generate a new image, *M*^(*C*)^, twice as large as *M*, that was initialized with zeros. Then, *M* was copied to *M*^(*C*)^ such that the centroid of *M* coincides with the center of *M*^(*C*)^ and *v*_1_ is aligned with the horizontal direction. The elements of *M*^(*C*)^ which were not overwritten with the elements of *M* remained zero.

#### Pair-wise distance measure between images

In this step a distance matrix (*D*(*l*, *m*)) was calculated whose entries are the pair-wise distances between images *l* and *m*. Two distance measures were employed: the Hellinger and Euclidean distances (*d*_*H*_)^27^,

3 $$D(l,\,m)={d}_{H}({M}_{l}^{(C)},\,{M}_{m}^{(C)})=\sqrt{2\,\sum _{i,j}\,{(\sqrt{\frac{{M}_{l}^{(C)}(i,j)}{{S}_{l}}}-\sqrt{\frac{{M}_{m}^{(C)}(i,j)}{{S}_{m}}})}^{2}}\,,$$

4 $$D(l,\,m)={d}_{E}({M}_{l}^{(C)},\,{M}_{m}^{(C)})=\sqrt{\sum _{i,j}\,{({M}_{l}^{(C)}(i,j)-{M}_{m}^{(C)}(i,j))}^{2}}.$$

#### Nonlinear dimensionality reduction

In order to extract meaningful information from the pair-wise distance matrix, *D*(*l*, *m*), we applied a nonlinear dimensionality reduction algorithm, directly to the pair-wise distance matrix. Two algorithms were tested: *IsoMap*^28^ and *t-SNE*^29^.

These algorithms assign, to each image in the database, a point in a n-dimensional space, whose coordinates will be referred to as mapped coordinates. While the algorithm also works with an arbitrary number of dimensions, in this paper a two dimensional space (*w*, *v*) is used for visualization reasons. The choice of a two dimensional space is appropriated because the residual variance (defined as in^28^) using two dimensions is of about 30%.

#### Computational runtime

All the described algorithms were implemented and run using MatLab in a portable computer with an Intel i7-7700HQ processor and 16 GB of RAM. We used the implementation of the nonlinear dimensionality reduction techniques written by van der Maaten *et al*.^30^. With this setup, it takes 5294 seconds (one and a half hours) to preprocess all the (1213) images including aligning and filtering, 1054 seconds (18 minutes) to compute the Hellinger distance matrix (735078 pair-wise distances), it takes 41 seconds for IsoMap to compute the mapping, and it takes 25 seconds for t-SNE to compute the mapping. It must be noted that all this runtimes could be significantly improved by rewriting the algorithms in a compiled language.

### Informed consent statement

Informed consent statements were obtained from all the participants of the study.

### Guidelines and regulations

OCT image acquisition and analysis were performed in accordance with the relevant European guidelines and regulations.
